# Scan-Chain-Fault Diagnosis Using Regressions in Cryptographic Chips for Wireless Sensor Networks

**DOI:** 10.3390/s20174771

**Published:** 2020-08-24

**Authors:** Hyunyul Lim, Minho Cheong, Sungho Kang

**Affiliations:** Electrical and Electronic Engineering Department, Yonsei University, Seoul 03722, Korea; lim8801@soc.yonsei.ac.kr (H.L.); cmh9292@soc.yonsei.ac.kr (M.C.)

**Keywords:** cryptography, wireless sensor networks, machine learning, scan-chain diagnosis

## Abstract

Scan structures, which are widely used in cryptographic circuits for wireless sensor networks applications, are essential for testing very-large-scale integration (VLSI) circuits. Faults in cryptographic circuits can be effectively screened out by improving testability and test coverage using a scan structure. Additionally, scan testing contributes to yield improvement by identifying fault locations. However, faults in circuits cannot be tested when a fault occurs in the scan structure. Moreover, various defects occurring early in the manufacturing process are expressed as faults of scan chains. Therefore, scan-chain diagnosis is crucial. However, it is difficult to obtain a sufficiently high diagnosis resolution and accuracy through the conventional scan-chain diagnosis. Therefore, this article proposes a novel scan-chain diagnosis method using regression and fan-in and fan-out filters that require shorter training and diagnosis times than existing scan-chain diagnoses do. The fan-in and fan-out filters, generated using a circuit logic structure, can highlight important features and remove unnecessary features from raw failure vectors, thereby converting the raw failure vectors to fan-in and fan-out vectors without compromising the diagnosis accuracy. Experimental results confirm that the proposed scan-chain-diagnosis method can efficiently provide higher resolutions and accuracies with shorter training and diagnosis times.

## 1. Introduction

Wireless sensor networks (WSNs) are composed of several sensor nodes that are deployed in the area to be monitored, through certain topologies and for certain purposes. Through some suitable methods and their respective information exchanges, WSNs collaboratively perceive physical world information and collect and collate the information of perceived objects within the network coverage area [1,2]. Because of these characteristics, WSNs have been widely used for various environmental, health, military, and commercial applications, such as intelligent transportation, smart homes, industrial monitoring, logistics, and healthcare systems [3,4]. However, the rapid deployment of WSNs has created critical problems in privacy and security [5,6,7]. Consequently, cryptography is generally implemented to ensure the security and integrity of information and data in WSNs. Cryptography systems can be divided into symmetric-key and asymmetric-key algorithms. Symmetric-key algorithms utilize only one secret key to cipher and decipher information, whereas asymmetric-key algorithms utilize public and secret keys to encrypt and decrypt information. The public key is freely available to anyone, whereas the private key is maintained secure and is only known to its owner. These cryptography algorithms require numerous modular multiplication and exponentiation operations, which are computationally expensive. Therefore, specific circuits for cryptographic algorithms that require high computational power have been designed [8].

Cryptographic circuits must be rigorously tested to guarantee the accuracy of cryptographic algorithms. Therefore, a scan structure is generally inserted to test cryptographic circuits. Scan structures are widely used in very-large-scale integration (VLSI) circuits as a design-for-test, as they increase the fault coverage and diagnosability by enhancing the controllability and observability of the digital circuit logic. The normal operation of scan chains in the circuits is guaranteed for accurate scan testing. However, if a fault occurs in scan chains, the test cannot be performed, and the yield decreases. In particular, several defects occurring in the initial manufacturing process are expressed as scan-chain faults. Therefore, the fault phenomenon propagates, widely reducing the accuracy of fault diagnosis. Hence, 10–30% of yield loss is caused by faults in the scan chain [9,10].

In addition, the cryptographic chips are generally protected by the secure scan-chain architecture to defense the scan channel attack. The secure technique generally uses the secret-key management policy to encrypt the scan-chain content during testing. It is required that inserting the encryption hardware composed of the internal registers in the scan-chain architectures. The secure scan architecture of the cryptographic circuit reduces controllability and observability [11,12], reducing the accuracy of scan-chain diagnosis. Consequently, scan-chain diagnosis has become a crucial issue in semiconductor manufacturing for cryptographic circuits.

Therefore, several scan-chain-diagnosis methods have been studied. Special-tester-based diagnostic methods [13,14,15,16] use a tester to control the scan operation and physical failure analysis equipment to identify the defective scan cells and locations of defective dies. These methods provide high resolution and accuracy. However, these special testers are expensive, and their operations are time consuming. Hardware-based diagnostic methods [17,18,19,20] modify a scan-chain structure or scan-cell design to enhance the diagnosability. They can easily identify a defective scan cell from a scan chain. However, these methods are not typically used due to additional hardware overheads and diagnostic complexity when a fault occurs in the additional control logic.

Finally, software-based diagnostic methods [21,22,23,24,25] apply algorithmic diagnosis by analyzing the observed responses of the patterns, which comprise shift-in, capture, and shift-out operations, from all scan chains. Such software-based scan-chain diagnoses are typically used because they can increase the yield of manufacturing without requiring additional hardware overheads and costs for special testers. However, these methods do not provide satisfactory results for scan-chain diagnosis. They require a significant amount of fault simulations to search for candidate scan cells in a failure case. Therefore, high computation power is required for executing several fault simulations, which becomes time-consuming for a scan chain with numerous cells. Furthermore, these methods cannot achieve sufficient resolution and accuracy.

Machine learning has been widely used in the field of fault diagnosis [26,27,28,29,30] as well as in scan-chain diagnosis to achieve sufficient resolution and accuracy [31,32,33]. For example, in [33], multistage artificial neural networks (ANNs) were used to diagnose scan-chain faults. A coarse global neural network (CGNN) was used to select several suspected scan cells (affine group) from among all scan-chain cells, and a refined local neural network (RLNN) was used to identify the final suspected scan cell in the affine group. The CGNN used a vector called an integer failure vector (IFV), a bitwise summation of all the binary failure vectors, as training and inference. In addition, the RLNN was trained using a cascaded failure vector, which was created by concatenating all the binary failure vectors. The use of such ANNs for scan-chain diagnosis was effective in improving the resolution and accuracy. However, multistage ANNs have some shortcomings due to their characteristics. First, several ANNs are required for the inference: one CGNN for each scan chain and several RLNNs for each affine group on only one scan chain. Moreover, the vector used for the RLNN is extremely long; hence, the network size is increased. Therefore, multistage ANNs require significant amounts of training time.

Therefore, this article proposes a novel scan-chain diagnosis method involving regressions and various filters. The controllability and observability from the logic circuit structure are applied to the fan-in and fan-out filters; thus, the fault-affected and fault-affecting cell information from the capture sequence are collected by the fan-in and fan-out filters, respectively. Therefore, raw failure vectors are compressed by applying filters, and the values with fault effects remain only at the new vectors. This can reduce the number of models for a chain and the number of dimensions of the models without losing the resolution and accuracy of scan-chain diagnosis. Therefore, the proposed regression reduces the training and diagnosis times as well as improves the resolution and accuracy of scan-chain diagnosis.

The remainder of this article is organized as follows. The motivations for introducing the fan-in and fan-out filters are presented in Section 2. The regressions and filters are described in Section 3. The simulation results are presented in Section 4. Finally, the conclusions are provided in Section 5.

## 2. Motivations

In this section, the motivations for introducing the fan-in and fan-out filters are presented. First, a software-based scan-chain diagnosis is described in Section 2.1. Second, the motivation for using machine-learning algorithms in scan-chain diagnosis is described in Section 2.2. Subsequently, the concept of sensitive scan cells is presented in Section 2.3.

### 2.1. Software-Based Scan-Chain Diagnosis

Software-based diagnostic methods [21,22,23,24,25] apply software-based algorithms to identify faulty cells. Such diagnosis uses test patterns to diagnose scan-chain faults. Generally, these test patterns can be categorized into the following three categories:

1. A chain pattern: It comprises only shift-in and shift-out, without a single capture process. This pattern aims at verifying whether the chain fails, and it can categorize various scan-chain defects into three common types—the stuck-at (SA) fault (stuck-at 1, stuck-at 0), slow (slow-to-rise, slow-to-fall, slow), and fast (fast-to-rise, fast-to-fall, fast) types.
2. A scan automatic test pattern generation (ATPG) pattern: It comprises shift-in, shift-out, and multiple-capture processes. The purpose of this pattern is to test the circuit logic.
3. A special chain diagnostic pattern: It is generated solely for scan-chain diagnosis.

The software-based diagnostic methods use three test patterns and failure logs to diagnose scan-chain faults. First, a flush test is conducted to find the defective scan chain. Next, a fault is injected in each scan cell, and a fault simulation is executed for each cell to collect its simulated fault response in a software-based simulation environment. Subsequently, the capture process of the loaded values (test stimulus) is simulated, and the simulated captured values are compared with the observed values (test response). After executing all the simulations, the cell with the best match is selected as the suspected scan cell. If it cannot be identified in the defective chain, special chain diagnostic patterns are used to obtain a higher resolution and accuracy.

**Figure 1:**
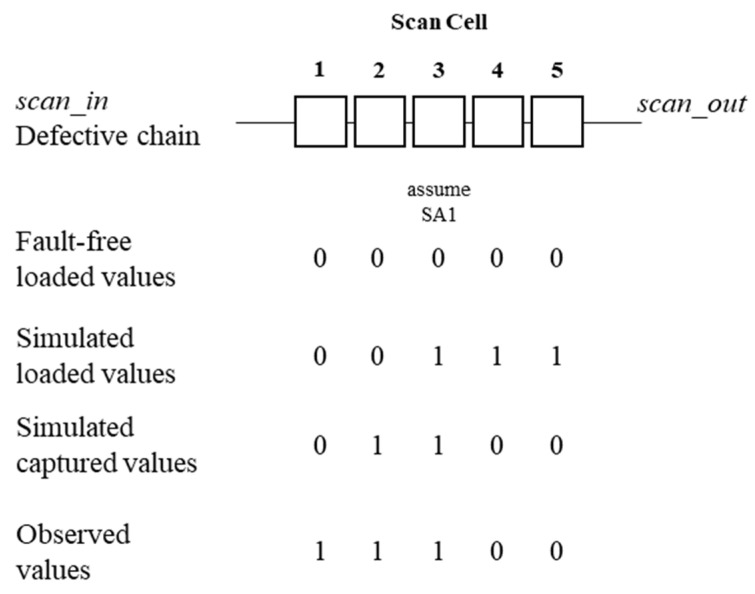
Example of software-based scan-chain diagnosis.

Figure 1 illustrates an example to explain the software-based scan-chain diagnosis. To simplify the explanation, each scan cell is indexed from the scan-out to the scan-in cells. The scan cells between the scan-in and scan cells are called the upstream scan cells, and those between the scan-out and scan cells are called the downstream scan cells. Assume that a stuck-at-1 (SA1) fault exists in Cell 2 in the defective chain.

First, all the simulated loaded values of the defective chain are 0s. Next, the simulated loaded values are changed to (0 0 1 1 1) because the test stimulus values of the upstream scan cells of the defective cell, Cell 3, are polluted by the defective cell during the shift sequence. After the fault simulation, the simulated captured values of the defective chain are (0 1 1 0 0). Subsequently, suppose the observed values on the automatic test equipment (ATE) are (1 1 1 0 0). The observed values of the downstream scan cells of the defective cells are polluted by the defective cell during the shifting sequence. The observed value at Cell 1 should be polluted by the SA1 fault. Thus, the captured value on the ATE can be (0 1 1 0 0). Therefore, the SA1 fault may be in Cell 3. Furthermore, the lower bound of the defective chain is at Cell 3 because a 0 value cannot be observed at the lower bound of the SA1 fault cell.

The software-based algorithms run the fault simulation as described above for all test patterns. The candidate cells are determined from the lower and upper bounds of the defective chain. Then, the scores are calculated by comparing the observed and simulated observed values of each candidate cell, thereby confirming the location of the defective cell. However, these methods do not provide satisfactory results for intermittent faults. The intermittent fault diagnosis accuracy of the simulation-based scan-chain diagnosis using manufacturing test patterns is 52% on the target circuit [34]. In addition, the accuracy of the simulation-based scan-chain diagnosis using signal profiling is 57% [35]. Furthermore, these methods cannot achieve sufficient accuracy.

### 2.2. Machine Learning for Fault Diagnosis

Machine learning is an effective technique that enables systems to learn automatically from experience without being explicitly programmed. It has been applied in various fields, such as speech recognition, robotics, medical diagnosis, and computer vision to solve numerous problems [28]. The main idea of machine learning is to train a machine through example data to solve a cognitive task.

**Figure 2:**
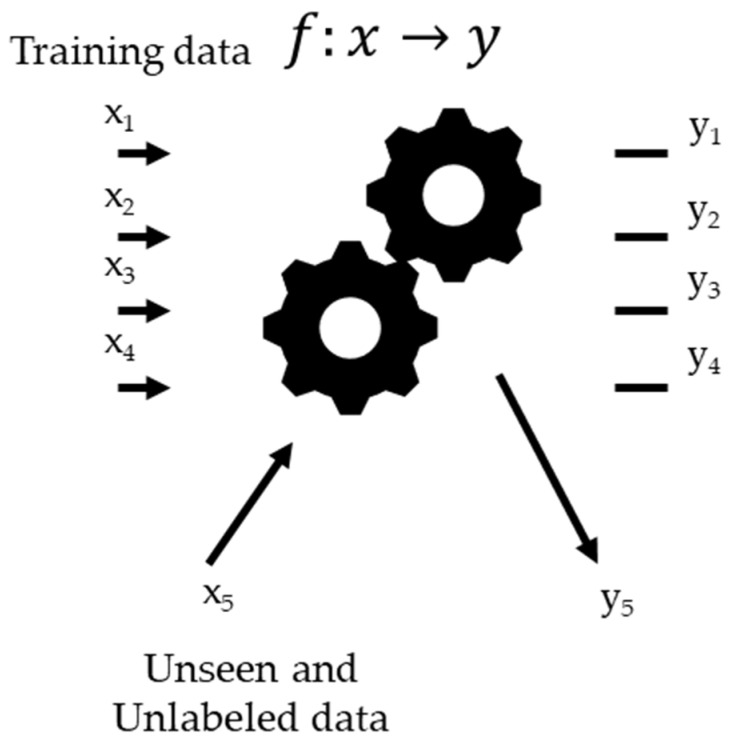
Scope of machine learning.

Machine learning is expressed by a function f:x →y  that has an unknown intricate closed-form mathematical expression, as shown in Figure 2. The problems encountered when using machine learning are expressed as a function *f* using *N* datasets (*x_i_*, *y_i_*), *i* = 1, 2, 3, ∙∙∙, *N*. In machine learning, *x_i_* is called a feature, and *y_i_* is called a target. By learning these datasets, machine learning can be used to create a function *f* that can infer a correct target even when an unseen feature is generated. Because of the characteristics of training and inference, machine learning can be applied in several fields where a large volume of data is available to train the models.

Scan-chain diagnosis is also suitable in terms of machine learning because a considerable number of labeled failure log datasets are generated during manufacturing. However, machine learning might not operate as intended if training is performed with only raw data. Although machine learning appears to be an all-round contributor in big-data processing, the intended results can only be achieved by understanding the problem accurately. For a specified target, some domain knowledge and decision-making processes are required to select a few effective features from among several possible features.

Owing to the recent proliferation of machine learning, various machine-learning techniques have been used for scan-chain diagnosis. However, previous scan-chain diagnosis methods using ANNs and unsupervised machine learning [31,32,33] merely train simple ANNs based on raw failure log datasets. Therefore, the input vector is long, and several ANNs must be used in only one scan chain; this results in long training diagnosis times. However, if the effects of the fault are compressed by specific filters generated based on the characteristics of the circuit structure, the fault can be diagnosed using a short vector.

### 2.3. Sensitive Cells in Scan-Chain Diagnosis

**Figure 3:**
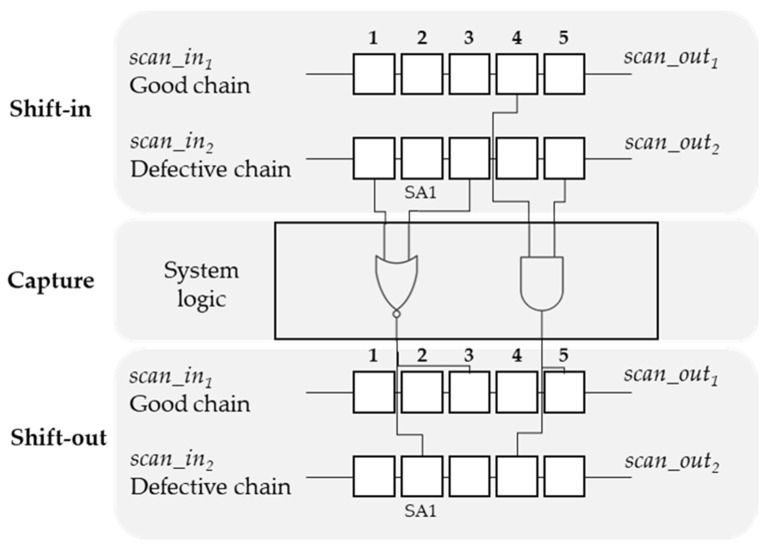
Sensitive cells in scan testing.

In the general logic scan test process, test patterns are shifted into the circuit logic and shifted out to the scan-chain cells as a response in the capture process. Therefore, if an error is injected in a scan cell in the scan shift process, it can be spread to other cells through the circuit logic. Hence, errors will occur at the connected cells through the circuit logic. These connected cells of the scan cell are called sensitive cells and are classified into fan-in and fan-out cells. The fan-in cells affect the target cell, whereas the fan-out cells are affected by the target cells. For example, as shown in Figure 3, the circuit contains two scan chains, i.e., 1 and 2, with five scan cells from Cell 1 to Cell 5. Assume that an SA1 fault occurs in Cell 2. Cell 3 of the defective chain is connected to Cell 3 of the fault-free chain and Cell 2 of the defective chain through a NOR gate. Therefore, if an error is injected into Cell 3 of the defective chain from the shift process, it is highly likely that an error occurs in Cell 3 of the fault-free chain and Cell 2 of the defective chain after the capture process. Therefore, Cell 3 of the fault-free chain and Cell 2 of the defective chain are the fan-out cells of Cell 3 of the defective chain, thereby making Cell 3 of the defective chain the fan-in cell of Cell 3 of the fault-free chain and Cell 2 of the defective chain.

The concept of a sensitive cell has been studied previously. Scan-chain reordering [36,37] and stitching [38] have been proposed to consider logic dependency and controllability between scan cells, where scan-chain diagnosis was improved using a circuit structure. In particular, using the fan-in and fan-out dependencies between scan cells, these methods distributed sensitive cells to other chains, thereby obtaining more clues from failure logs. Therefore, software-based scan-chain diagnosis, such as full-masking scan-chain diagnosis, achieved a higher accuracy on reordered scan chains based on sensitive cells than the conventional scan-chain diagnosis.

This concept of sensitive cells can be used for scan-chain diagnosis using ANNs as well. As the impact of a fault in each cell spreads to the sensitive cells of each cell, filters that can contain this impact from the sensitive cells can be used to generate reduced input vectors without compromising accuracy. Therefore, if a raw failure vector is edited using these filters, a shorter input vector can be constructed for the ANN to diagnose a fault with sufficient accuracy.

## 3. Proposed Methodology

In this section, the key idea of the study is presented. First, the overall flow of the proposed method and the dataset preparation are described in Section 3.1. Section 3.2 describes the fan-in and fan-out filters that can compress the raw failure log to shorter vectors by highlighting the tendency of the failure. Finally, the selection of training and loss functions is described in Section 3.2.3.

### 3.1. Overall Flow and Dataset Preparation

**Figure 4:**
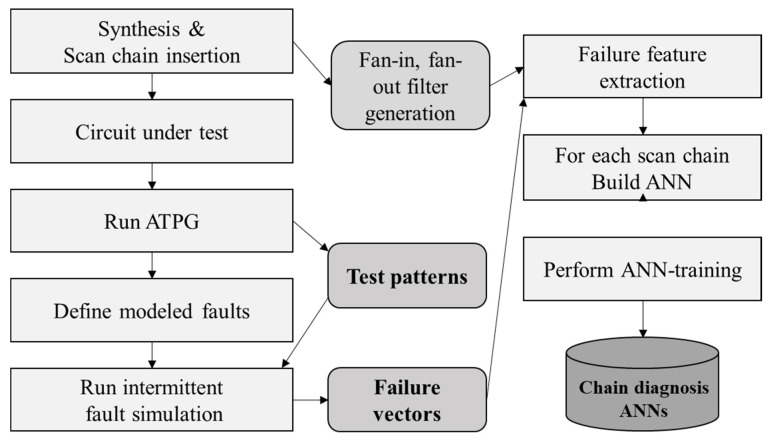
Flowchart of the proposed methodology. ATPG: automatic test pattern generation; ANN: artificial neural networks.

Figure 4 shows the overall flowchart of the proposed methodology. The left-hand side of the flowchart shows the test pattern generation process in which failure log datasets are generated on the generated test patterns. A target circuit is synthesized, and scan chains are inserted into the target circuit using a Synopsys design compiler. Next, an ATPG is executed on the scan-chain insertion circuit under test. Subsequently, the test patterns for the target circuit are produced, and failure log datasets are generated to obtain the training and test datasets for the target circuit. Moreover, a fault model is defined similar to the SA fault, slow, and fast models. Furthermore, an intermittent fault simulation is executed to produce the failure log datasets of the defined fault model.

The right-hand side of the flowchart shows the failure feature vector generation process in which the ANN of each scan chain is constructed with the failure feature vectors. First, during test pattern generation, a hierarchical cell report and scan-chain report are generated, and thereby, fan-in and fan-out filters are generated. Next, failure features are extracted using the failure log datasets, fan-in filters, and fan-out filters, to generate failure feature vectors. Subsequently, ANNs are trained for each chain using the failure feature vectors. Finally, the scan-chain diagnosis using the ANNs infer the defective scan cells in each scan chain.

The failure log datasets—comprising failure logs and output labels (the position of the defective cell)—are generated using the following three parameters: (1) fault model, (2) fault location, and (3) fault probability.

1. Fault model: A modeled fault that is one of the following fault types: SA0, SA1, slow-to-rise, slow-to-fall, fast-to-rise, fast-to-fall, fast, or slow.
2. Fault location: A modeled fault is generally assumed to occur in the input wire or output wire of the scan cells or scan cell logic. Therefore, the output of the scan cell can be affected by the modeled faults. The fault location is the location of the scan cell, and it is labeled by the chain and cell numbers. For example, if the third cell in the second chain has a fault, the output label becomes (2, 3).
3. Fault probability: A modeled fault may occur during the processing time. We determined the probability of the fault occurring during the generation of the failure log dataset. For example, assume that a scan chain has seven cells. If the probability of an SA0 fault occurring in Cell 3 is 20%, then each test stimulus at Cells 4–7 may have a 20% probability of failure. By contrast, the test response of Cells 1, 2, and 3 may have a 20% probability of failure.

The fault probability is determined as 10%, 20%, 30%, 40%, 50%, 60%, 70%, 80%, 90%, and 100%. Depending on the probability, the failures are injected in the test stimulus and the test response, as shown below.

1. Perform ATPG and obtain the standard test interface language (STIL) file that contains the test patterns of the target circuit.
2. Inject errors to the test stimulus of the target scan chain with the determined fault probability.
3. Perform fault simulation with the failure-injected STIL file and obtain the failure log datasets through the errors in the test stimulus.
4. Inject errors in the test response of the target chain with the determined fault probability.

In the failure log datasets, various failure cases can be generated even for the same probability. Hence, various vectors can be obtained in the same group of probabilities through 10 iterations.

### 3.2. Failure Feature Extraction

There are two kinds of failures due to the scan-chain faults: test stimulus failure and test response failure. The test stimulus failures are the polluted values of the test stimulus during the shift sequence. Because the value of the test stimulus is loaded serially into the scan chain through a scan-in port-by-shift sequence, the test stimulus values of upstream scan cells of the defective cell are polluted by the defective cell during the shift sequence. This test stimulus failure can cause multiple failures in the fault-free chain through the connected combinational circuits during the capture sequence. Meanwhile, the test response failures are the failures in the test response of the defective chain during shift sequence. In the shift sequence, the state of the scan chain by the capture sequence (test response) is shifted out through scan-out port. Therefore, the test response values of the downstream cells of the defective cell are polluted by the defective cell during the shift sequence. This test response failure can cause only one failure. Therefore, test stimulus failure can cause more failures than test response failure.

Therefore, a fault that occurs near the scan-in cell can cause more test stimulus failures than the test response failures, increasing the number of failures in the failure log. On the other hand, a fault that occurs near the scan-out cell can cause fewer errors in the test stimulus and increase the number of errors in the test response of the defective scan chain, decreasing the number of failures in the failure log.

**Figure 5:**
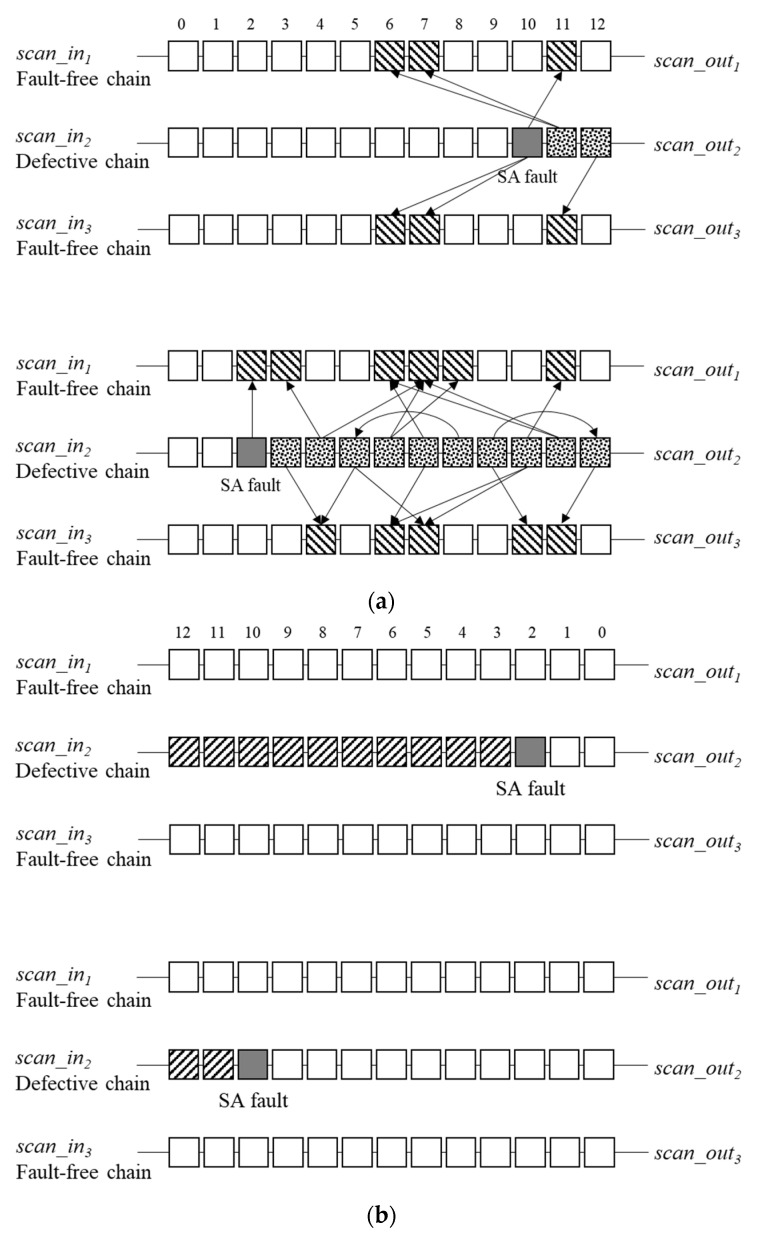
Failure tendencies: (**a**) failure tendency caused by shift-in; (**b**) failure tendency caused by shift-out.

Figure 5 shows some relevant examples. To simplify the explanation, defective cells are marked by gray boxes; the cells in which errors occur at the test stimulus are marked by the dot patterned boxes; the cells in which failure is observed at the test response are marked by diagonally lined boxes. As shown in the upper part of Figure 5a, assume that an SA fault exists in Cell 10 in the defective chain. This SA fault will affect the test stimulus of Cells 11 and 12 in the defective chain. Therefore, errors will only spread to sensitive Cells 10, 11, and 12 in the fault-free chains. In contrast, assume that an SA fault exists in Cell 2 in the defective chain, as illustrated at the bottom of Figure 5a. This SA fault affects the test stimulus in Cells 2–12 in the defective chain. Hence, several more errors appear in the fault-free chains at the scan output compared with the case in Cell 10. However, the number of failures in the defective chain decreases as the defective scan cell approaches the scan-in cell and increases as the defective scan cell approaches the scan-out cell, as shown in Figure 5b.

These effects are more prominent in the sensitive cells of each cell. Therefore, a shorter vector that contains all the failure information would be generated by vectorizing the tendencies of the failures based on an investigation of all the sensitive cells in each chain. Consequently, filters are necessary to generate a vector that can train these tendencies from the raw failure log datasets.

The sensitive cells are categorized into two types. The first type includes the fan-out cells, which are the affected cells in all the chains that are connected to the cell of the defective chain. Therefore, if an error occurs at a cell, the observed responses of one or more of the fan-out cells will contain errors. For example, as shown in Figure 3, if an error occurs in the test stimulus at Cell 0 in Chain 1, the test response of Cell 0 in Chain 1 and that of Cell 1 in Chain 2 may lead to failures in these cells. Therefore, Cell 0 and Cell 1 in Chain 2 are the fan-out cells of Cell 0 in Chain 1, in contrast to the fan-in cells. Similar to this example, the fan-out filters identify the fan-out cells in the defective chain.

The second type is the fan-in cells, which are the affected scan cells at the defective chain that are connected to the cell of the fault-free chain. If there is an error in the response of a cell in a chain other than the detective chain, this error must have originated from the error stimulus of a cell in the defective chain. The fan-in cells are the cells observed by backtracking the cells that are connected. Therefore, an error occurring at a cell results in errors in the test stimulus of one or more of the fan-in cells of the cell. For example, Cell 1 in Chain 1 and Cell 0 in Chain 2 are connected to an AND gate cell, which is connected to Cell 0 in Chain 1 and Cell 1 in Chain 2. Consequently, Cell 1 in Chain 1 and Cell 0 in Chain 2 are the fan-in cells of Cell 0 in Chain 1 and Cell 1 in Chain 2.

The fan-in and fan-out filters are generated by analyzing the circuit logic structure to extract the failure feature from the failure logs. First, the hierarchical cell and scan-chain reports are analyzed. The hierarchical cell report details the connection of each cell, such as the input pins, output pins, net driver pins, and net load pins, whereas the scan-chain report shows the name of the scan cell belonging to each scan chain. Accordingly, the fan-in and fan-out filters are generated by searching all the fan-in and fan-out cells of each cell.

#### 3.2.1. Fan-In Filters

The fan-in filter accumulates the number of errors affected by the fan-in cells in the defective chain. Hence, each element of a vector reflects the number of errors affected by each cell in the defective chain. Therefore, the length of the fan-in vector is the same as that of the defective chain. First, the fan-in filters detect the fan-in cells of all the scan cells in the defective chain. Subsequently, the number of errors in the fan-in cells of each cell is applied to the element reflecting the cell of the fan-in vector.

To apply the number of errors, failure log datasets are analyzed. If an error exists at the location of a cell in the failure log dataset, this information is applied in the elements of the fan-in cells in the fan-in vectors, if no error occurs in the test response of the fan-in cells. Because, if a failure occurs on the test stimulus of the defective chain, failure does not occur again on the test response of the chain except the defective cell. Therefore, each element of fan-in vectors reflects the number of errors in the fault-free chain caused by each cell in the defective chain. This operation is performed for all failure log datasets. By adding all vectors investigated in this manner, a fan-in vector is created.

**Figure 6:**
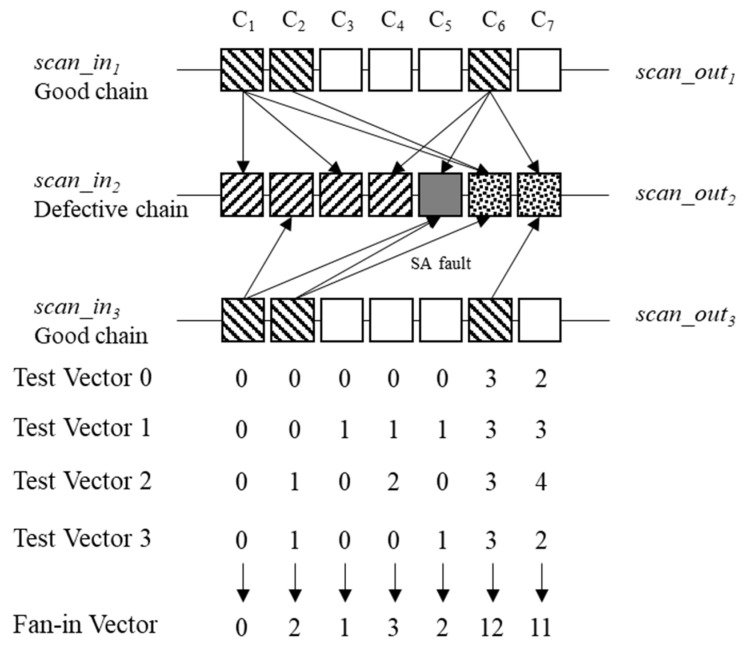
Example of fan-in filter.

An example of the fan-in filters is demonstrated in Figure 6 and Table 1. The target circuit comprises three scan chains, each containing seven cells. For ease of explanation, Cell B in Chain A is referred to as S_A_C_B_. If an SA0 fault occurs at Cell 5 in scan chain 2 (S_2_C_5_), then errors occur at Cells 1, 2, and 6 in scan chain 1 (S_1_C_1_, S_1_C_2_, S_1_C_6_), Cells 1–5 in scan chain 2 (S_2_C_1_, S_2_C_2_, S_2_C_3_, S_2_C_4_, S_2_C_5_), and Cells 1, 2, and 6 in scan chain 3 (S_3_C_1_, S_3_C_2_, S_3_C_6_) in the observed responses of test pattern 0.

An analysis of the circuit structure in Table 1 shows that S_1_C_1_ is affected by S_2_C_1_, S_2_C_3_, and S_2_C_6_; S_1_C_2_ is affected by S_2_C_5_; S_1_C_6_ is affected by S_2_C_4_, S_2_C_5_, and S_2_C_7_; S_3_C_1_ is affected by S_2_C_2_ and S_2_C_5_; S_3_C_1_ is affected by S_2_C_5_ and S_2_C_6_; S_3_C_2_ is affected by S_2_C_7_. In pattern 0, the failure at the S_1_C_1_ is applied to the fan-in vector of S_2_C_1_, S_2_C_3_, and S_2_C_6_. There are failures on the test responses of S_2_C_1_ and S_2_C_3_. Therefore, only the element of S_2_C_6_ counts, <0 0 0 0 0 1 0>. The failure of S_1_C_2_ is applied to S_2_C_6_, <0 0 0 0 0 2 0>. The failure of S_1_C_6_ is applied to S_2_C_7_, <0 0 0 0 0 2 1>. The failure of S_3_C_1_ is applied to S_2_C_2_ and S_2_C_5_, but there are failures on the test response of S_2_C_2_ and S_2_C_5_, <0 0 0 0 0 2 1>. The failure of S_3_C_2_ is applied to S_2_C_5_ and S_2_C_6_, but there is failure on the test response of S_2_C_5_, <0 0 0 0 0 3 1>. The failure of S_3_C_6_ is applied to S_2_C_7_, <0 0 0 0 0 3 2>. Therefore, the fan-in vector of scan chain 2 becomes <0 0 0 0 0 3 2>. This fan-in vector is generated through all the failure log datasets. Subsequently, test patterns 0, 1, 2, and 3 acquire vectors <0 0 0 0 0 3 2>, <0 0 1 1 1 3 3>, <0 1 0 2 0 3 4>, and <0 1 0 0 1 3 2>, respectively. By adding all the vectors, the fan-in vector becomes <0 2 1 3 2 12 11>.

#### 3.2.2. Fan-Out Filters

If a fault occurs near the scan-in cell, the test stimulus will contain more errors than when a fault occurs near the scan-out cell. Therefore, the failure in the test response would have spread more than that in the case where it is close to the scan-out cell.

Hence, the fan-out filter accumulates the number of errors at the fan-out cells in all the chains. Therefore, each element of a vector reflects the number of errors in the fan-out cells. Accordingly, the length of the fan-out vector is the same as the number of cells. First, the fan-out filters detect the fan-out cells of all the scan cells. Subsequently, the number of errors in the fan-out cells of each cell is applied to the element reflecting the cell of the fan-out vector.

To obtain the features from the failure log dataset, the fan-out filters track the spreading cells of the shift-out failures from all chains using a circuit structure similar to that of the fan-in filter. Resembling the fan-in vector, each element of a vector reflects the number of errors in the fan-out cells in the defective chain. The failure log datasets are analyzed to calculate the number of errors in the fan-out cells. The fan-out cells of each cell are searched, and the total number of errors that exist at all fan-out cells is accumulated in the elements of the cell in the fan-out vectors. Therefore, the element of the fan-out vectors reflecting a cell becomes the sum of the number of errors at all the fan-out cells of the cell. Moreover, all the failure log datasets are summed. This operation is performed for all the scan chains. Subsequently, a fan-out vector is created by adding all the vectors investigated thus.

**Figure 7:**
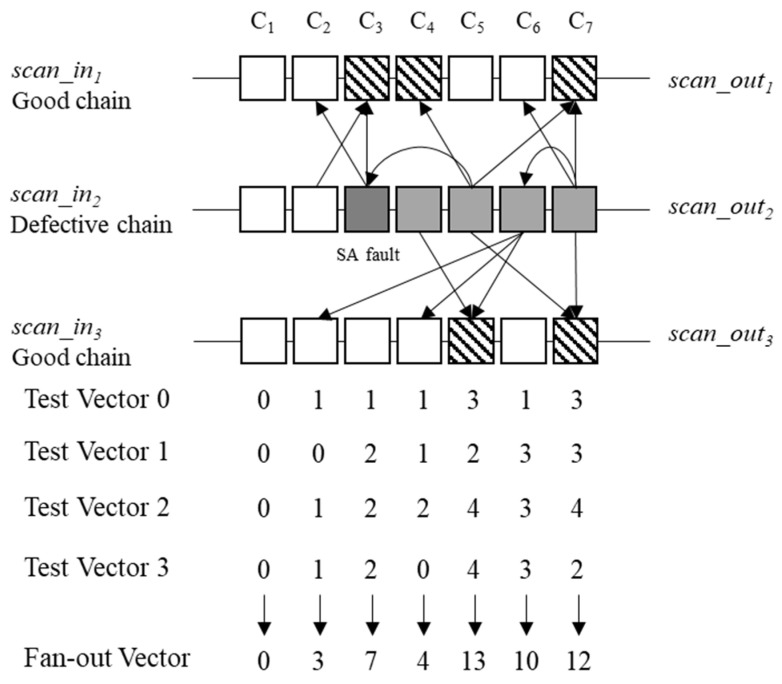
Example of the fan-out filter.

Figure 7 and Table 2 demonstrate the fan-out filters, where the target circuit comprises three scan chains, each containing seven cells. If an SA fault occurs at Cell 3 in scan chain 2 (S_2_C_3_), then test pattern 0 acquires the failure at Cells 3, 4, and 7 in scan chain 1 (S_1_C_3_, S_1_C_4_, S_1_C_7_); Cells 3, 4, 5, and 7 in scan chain 2 (S_2_C_3_, S_2_C_4_, S_2_C_5_, S_2_C_6_, S_2_C_7_); and Cells 5 and 7 in scan chain 3 (S_3_C_5_, S_3_C_7_).

In this circuit structure as shown in Table 2, S_2_C_2_ is affected by S_2_C_3_; S_2_C_3_ is affected by S_1_C_2_ and S_2_C_3_; S_2_C_4_ is affected by S_3_C_5_; S_2_C_5_ is affected by S_2_C_3_, S_1_C_4_, S_1_C_7_, and S_3_C_7_; S_2_C_6_ is affected by S_3_C_2_, S_3_C_4_, and S_3_C_5_; and S_2_C_7_ is affected by S_1_C_7_, S_2_C_7_, and S_3_C_7_. In this case, in pattern 0, the fan-out vector of S_2_C_1_ is 0 due to the absence of a fan-out cell in Cell 0; that of S_2_C_2_ and S_2_C_3_ is 1 due to failure at the fan-out cell S_2_C_3_; that of S_2_C_4_ is 1 due to failure at the fan-out cell S_3_C_5_. Finally, the fan-out vector of scan chain 2 becomes 0 1 1 1 3 1 3. These fan-out vectors are generated using all the failure log datasets. Subsequently, test patterns 0, 1, 2, and 3 acquire vectors <0 1 1 1 3 1 3>, <0 0 2 1 2 3 3>, <0 1 2 2 4 3 4>, and <0 1 3 0 5 3 2>, respectively. By adding all the vectors, the fan-out vector of scan chain 2 becomes <0 3 7 4 13 10 12>.

#### 3.2.3. Scan-Chain Diagnosis with Regressions

In this study, linear and logistic regressions were used for scan-chain diagnosis. The proposed scan-chain diagnosis requires only one trained model for the target chain and a target fault type, such as stuck-at 0, stuck-at 1, fast-to-rise, fast-to-fall, slow-to-rise, and slow-to-fall, to determine the accurate candidate of the scan-chain faults. With *N_c_* scan chains and *f* fault types, $N_{C}\times f$ models are trained to support the proposed scan-chain diagnosis.

**Figure 8:**
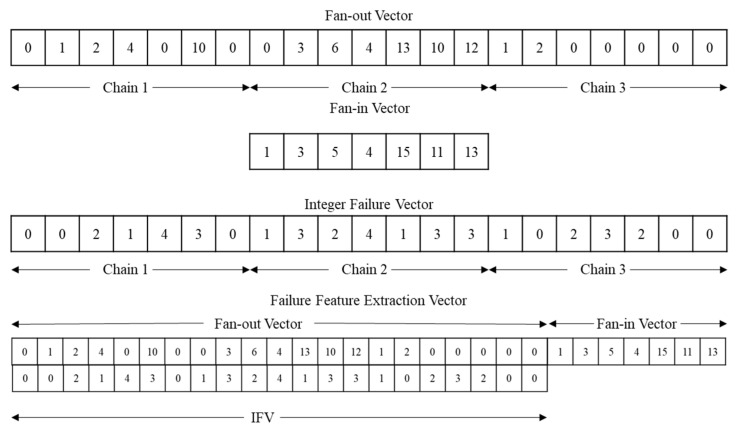
Example of failure feature vector.

The input vector of the proposed methodology, which is called the failure feature vector, is formed by combining three vectors. The first vector is the fan-in vector from the fan-in filter, the second vector is the fan-out vector from the fan-out filter, and the last vector is the IFV. These failure feature vectors are generated from the failure log datasets using the fan-in and fan-out filters. Furthermore, the generated failure feature vectors are categorized into train and test sets for training and testing, respectively. An example of the training vector is illustrated in Figure 8.

Linear regression and logistic regression were used as the machine-learning models through scikit-learn [39]. Linear regression analysis involves quantitatively determining the relation between the D-dimensional vector-independent variable *x* and the corresponding scalar-dependent variable *y*.

The linear regression model involves obtaining a function f(x) that outputs a value $\hat{y}$ that is closest to the corresponding dependent variable *y* for the independent variable *x*, Equation (1).

(1) $$\hat{y}=f\left(x\right)\approx y$$

If the relation between *x* and *y* is the following linear function f(x), it is known as a linear regression function Equation (2).

(2) $$\hat{y}=w_{0}+\;w_{1}x_{1}+\;w_{2}x_{2}+\cdots +\;w_{D}x_{D},$$

where $w_{0}$, …, $w_{D}$ are the coefficients of function f(x) and the parameters of this linear regression model. We considered a linear regression for solving the single-fault problem. The failure log dataset of a scan-chain fault exhibits a failure distribution tendency, and a linear regression is a suitable algorithm for solving this problem.

**Figure 9:**
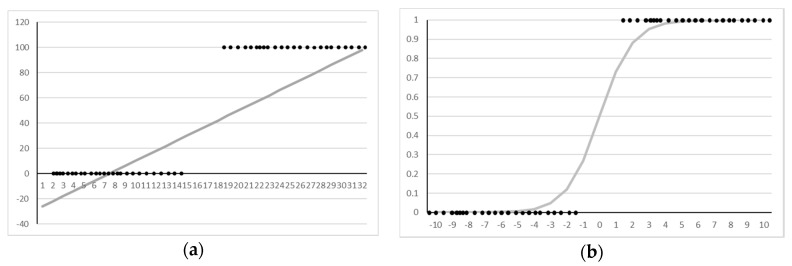
Pass/fail class with linear regression (**a**) and logistic regression (**b**).

The logistic regression is used to determine the probability of the existence of a certain class, such as pass/fail, win/lose, or healthy/sick. This scan-chain diagnosis problem can be categorized into a pass/fail class in the output element of each vector, which is the same as the logistic regression problem. This problem is difficult to solve because the output label is only 0/1 in the output vector, as shown in Figure 9a. Therefore, logistic regression is used to solve the pass/fail problem using logistic functions.

In several natural and social phenomena, the probability value for a specific variable often follows the form of an S-curve rather than a linear one. The logistic function expresses this S-curve as a function. Logistic functions can assume any value as an *x* value, but the output is always between 0 and 1. In other words, this function satisfies the requirement of a probability distribution function. The formula (3) is given below:

(3) $$y=\frac{1}{1+e^{-x}}$$

If the logistic function is used as an activation function in neural networks, we can easily solve the pass/fail problems using the neural networks, as shown in Figure 9b.

For a future multiple-fault problem, logistic regression is performed to solve a single scan-chain fault problem. If multiple faults occur in a scan-chain diagnosis, an output label cannot be obtained. Alternatively, a binary output label having the same length as that of the target scan chain is obtained. For algorithm optimization, scikit-learn can decide to use various solvers for logistic regression. The solvers used for scan-chain diagnosis are “A library for large linear classification (liblinear)”, “limited-memory Broyden–Fietcher–Goldfarb–Shanno (lbfgs)”, “stochastic average gradient (sag)”, and “saga”. Appropriate solvers for each training case are listed in Table 3.

The “liblinear” solver uses the coordinate descent algorithm. Hence, it successively solves the problem by performing an approximate minimization along the coordinate directions or hyperplanes. However, it may not be able to solve the nonstationary point or learn a multiclass model.

The “lbfgs” solver uses the Hessian matrix; however, it is approximated using updates specified by gradient evaluations. In addition, its limited memory stores merely a few vectors that represent the approximation implicitly. Therefore, if the training dataset is small, then “lbfgs” delivers the best performance, compared with the other methods. However, it may not converge if it is not safeguarded.

The “sag” solver uses optimization for the sum of a finite number of smooth convex functions. Therefore, its iteration cost is independent from the number of terms in the sum. However, it is faster than the other solvers for large datasets, as it incorporates the memory of previous gradient values when both the numbers of samples and features are large.

Additionally, the “saga” solver is a variant of the sag solver that supports the non-smooth option. This solver is for sparse multinomial logistic regression and is suitable for extremely large datasets, similar to the “sag” solver. An appropriate solver must be used to optimize the accuracy of scan-chain diagnosis. Therefore, the characteristics of the scan-chain failure data must be considered. As listed in Table 3, the size of the dataset is critical when selecting a solver. The size of the failure log datasets depends on the circuit size. Therefore, the solver for a circuit is selected based on the circuit size.

## 4. Experimental Results

The accuracy, training time, and diagnosis time of the proposed scan-chain diagnosis were evaluated through the experiments. The ITC’99 benchmark, which represents a wide range of industry-representative circuits and was used in this study, openrisc1200 (OR1200) and advanced encryption standard (AES) circuit are summarized in Table 4 [40].

Table 4 summarizes the characteristics of benchmark circuits. In the first and second columns, the circuit and gate count denote the circuit name and number of gates, respectively. The third and fourth columns show the numbers of scan cells and chains in the circuit, respectively. The fifth and sixth columns denote the maximum and minimum numbers of fan-in and fan-out cells in the circuit, respectively; the maximum number is shown on the left side of the slash (/), whereas the minimum number is shown on the right side of the slash (/).

The specifications of the computer used in the experiment are as follows:

CPU: Intel Core i7-770 @ 3.60 GHz
RAM: 64 GB

The scikit-learn solver does not use a graphics processing unit (GPU). Therefore, a GPU was not used. The datasets were generated with the probabilities of 10%, 20%, 30%, 40%, 50%, 60%, 70%, 80%, 90%, and 100%. The training and test datasets were generated through 10 iterations. The training and test data were obtained by splitting the datasets into 75% and 25%, respectively, using the train_test_split function in scikit-learn.

### 4.1. Scan-Chain Diagnosis Accuracy

The accuracy of scan-chain diagnosis was evaluated using the accuracy classification score. The results of the test data using our trained regressions were compared with the actual injected fault location. If the prediction results of our models are the same as those of the injected fault location, they are treated as successful cases; otherwise, they are considered unsuccessful. The percentage of successful cases in the total test cases represents the accuracy of the scan-chain diagnosis.

The accuracy of the scan-chain diagnosis of the intermittent SA0 faults occurring in the first scan chain is reported. For the training and test data, we attempted to generate as many cases as possible. Hence, after targeting each fault location and setting the probability of faults, a failure was generated several times to create various failure cases.

For example, as shown in Figure 7, the fault location was C_3_ in the defective scan chain. Hence, a failure may occur in the test stimuli at C_4_, C_5_, C_6_, and C_7_. These test stimulus vector elements and the test response vector elements where failure can occur are known as possible failure points. The failure generator generates a random number from 0 to 99 at each possible failure point. Subsequently, if the generated random number is higher than the probability value, such as 10, 20, …, 100, a failure is injected at that point; otherwise, a failure is not injected at that point. Therefore, each failure is injected at the test stimuli C_3_, C_4_, C_5_, C_6_, and C_7_ and at the test responses C_1_, C_2_, and C_3_ if each generated random number is higher than the probability value. Consequently, even if the probability and fault location are the same, different failure log datasets are generated in each iteration.

The results are presented in Table 5. The Tessent tool [31] indicated that the scan-chain diagnosis had a 72.43% accuracy. In addition, the scan-chain diagnosis using multistage ANNs [33] achieved an average accuracy of 86.98%, as shown in the labeled column [33]. The accuracy of the proposed scan-chain diagnosis was improved to 90.15% through logistic regression, which implies that its accuracy was increased by 3.17%, compared with that achieved in [33].

In addition, the open-source cryptography circuits, AES [41], and the open source core OR1200 [42] have been used to evaluate the performance of the proposed scan-chain diagnosis in the cryptographic circuits and the processor. “lbfgs” solver has been selected to optimize the logistic regression model of AES and OR1200. Subsequently, the scan-chain diagnosis accuracy of AES and OR1200 are 90.06% and 82.36%, respectively. However, it can be increased by changing the configuration of the logistic regression.

### 4.2. Training Time and Diagnosis Time

Table 6 presents the construction times for the scan-chain diagnosis of a target chain. Compared with the previous scan-chain diagnosis [33], the proposed diagnosis additionally requires the filter generation time (Filter Gen. Time). However, as shown in Table 6, the filter generation time is shorter than the training time. Therefore, it does not affect the construction time.

Let n be the number of training samples and d be the dimensionality of the features. The required training time is generally *O*(*nd*^2^). The dimensionalities of the features of the previous scan-chain diagnosis (*D_M_*) [33] and the proposed scan-chain diagnosis (*D_P_*) are defined as follows:

(4) $$D_{M}=SC_{total}\;\times N_{test\;patterns}$$

(5) $$D_{P}=\left(2\times SC_{total}\right)+SC_{defective}$$

where *SC_total_* is the number of scan cells, *N_test patterns_* is the number of test patterns, and *SC_defective_* is the number of scan cells in the defective chain. The proposed method reduces the dimensionality of the features from *D_M_* to *D_P_*.

Therefore, the training time required by the proposed scan-chain diagnosis is shorter than that required by the previous scan-chain diagnosis [33], as presented in Table 6. Moreover, with the increase in the circuit size, the previous scan-chain diagnosis becomes slower due to the requirement of multiple ANNs even for a single scan chain.

Therefore, the proposed scan-chain diagnosis reduced the construction time by more than 79%, compared with the previous scan-chain diagnosis. The diagnosis time, i.e., the inference time of all the test cases, is listed in Table 7. The proposed scan-chain diagnosis is significantly faster as it uses one model for one chain, whereas the previous scan-chain diagnosis [33] uses multiple ANNs for one chain. As the sizes of the circuit and layers increased, the diagnosis time of the proposed scan-chain diagnosis slightly increased. Moreover, the diagnosis time of the proposed scan-chain diagnosis was reduced by more than 99.9984%, compared with that of the previous diagnosis. The results of experiment in the cryptographic circuit, AES are also added in Table 6 and Table 7. The construction time and diagnosis time of AES are 0.18 h and 3.9 microseconds, respectively.

In addition, the results in the processor, OR1200 are included in Table 6 and Table 7. Because the number of scan cell of the circuit is increased to 3420, the construction time and diagnosis time are increased to 9.05 h and 45.77 microseconds, respectively.

The reduction in training and diagnosis times facilitates the application of scan-chain diagnosis in semiconductor industries. As the training and diagnosis times are critical to satisfy the time-to-market goal for industrial circuits, the proposed scan-chain diagnosis is suitable, as its training time is 50% of that of the previous diagnosis. Therefore, the preparation time for diagnosing industrial circuits reduces to 79.80% of that of the previous methods. In addition, the proposed scan-chain diagnosis enables the time-to-market goal to be achieved by improving the diagnosis speed in the semiconductor industry, where several cases require diagnosis.

The training time, diagnosis time, and accuracy of scan-chain diagnosis of logistic regression change depend on the solvers. Moreover, the regularization strength affects the accuracy of logistic regression. The regularization strength refers to a penalty to increase the magnitude of parameter values to reduce overfitting. Hence, we measured the accuracy of scan-chain diagnosis, training time, and diagnosis time of B12 while changing the solver and the inverse of the regularization strength of the logistic regression.

**Figure 10:**
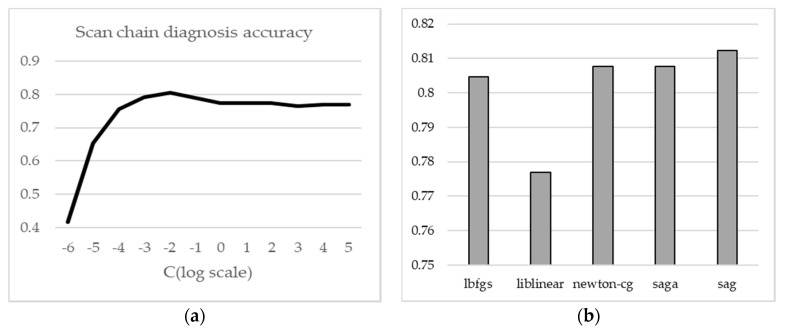
Accuracy of scan-chain diagnosis depending on regularization strength value and solvers on B12: (**a**) accuracy of scan-chain diagnosis depending on the regularization strength value (C); (**b**) accuracy of scan-chain diagnosis with various solvers at the inverse of regularization strength value e^−2^.

Figure 10 shows the accuracy of scan-chain diagnosis depending on the inverse of the regularization strength with the lbfgs solver. Moreover, the inverse of the regularization strength was changed to a logarithmic scale (i.e., 0.01, 0.1, 1, 10, 10^2^ …). The accuracy increases as the inverse of the regularization strength increases from $1\times e^{-6}$ to $1\times e^{-2}$. Subsequently, the accuracy gradually decreases as the inverse of the regularization strength increases. Therefore, the accuracy of scan-chain diagnosis is the highest when the inverse of regularization strength is $1\times e^{-2}$. Figure 10a demonstrates the impact of the inverse of the regularization strength on the accuracy.

Additionally, we measured the accuracy of scan-chain diagnosis by changing the solvers with a fixed inverse of the regularization strength, $1\times e^{-2}$. The accuracy varies slightly depending on the solver. However, this difference is small compared with the difference according to the inverse of the regularization strength; hence, if the appropriate regularization strength is determined, the solver does not significantly affect the scan-chain diagnosis.

### 4.3. Accuracy through Failure Log Dataset Property

The correlation between the circuit characteristics and accuracy was analyzed to improve the accuracy of the scan-chain diagnosis. For the analysis, B12 was used owing to its significantly lower training time than those of other circuits, with a comparable accuracy of scan-chain diagnosis.

First, the accuracy was analyzed according to the location of each cell in the scan chain by predicting the test stimulus. This model was trained to determine whether it can predict the test stimulus from the failure log dataset. Therefore, only logistic regression, specifically multiclass logistic regression (OneVsRest-Classification), was used. In this training, the input vector was a binary failure vector, and the output label was a test stimulus failure vector. The binary failure vectors were obtained from the failure log dataset. The total binary failure vector was divided among all test patterns, namely, test pattern 0, test pattern 1, … test pattern N. The binary failure vector that matched each test pattern was the input vector. In addition, the test stimulus failure vectors were obtained during the failure generating sequence. Resembling the scan-chain diagnosis, the training data and test data were obtained from 75% and 25% of the dataset, respectively, using the *train_test_split* function in scikit-learn.

As shown in Figure 6, a fault occurred in C_3_ in the target scan chain. Consequently, failures occurred at the test stimuli C_3_, C_4_, C_5_, and C_7_. Hence, the test stimulus failure vector was <0 0 1 1 1 0 1>. Additionally, the binary failure vectors from this test pattern were <0 0 1 1 0 0 1>, <1 1 0 0 0 1 0>, and <0 0 0 0 1 0 1>. These models predicted the test stimulus failure vector from a cascaded set of binary failure vectors, known as a cascaded binary vector. Accordingly, the input vector was <0 0 1 1 0 0 1 1 1 0 0 0 1 0 0 0 0 0 1 0 1>, and the output label was <0 0 1 1 1 0 1>. In the prediction, the incorrect case was obtained by comparing the prediction and output labels. If the obtained prediction label is <0 1 1 1 1 0 1>, then the output of Cell 1 is incorrect. In this case, the accuracy of Cell 1 is reduced.

Table 8 lists the fan-in and fan-out characteristics and the accuracy of scan-chain diagnosis of each cell. The first and second columns show the cell number and the cell prediction accuracy of each scan cell, respectively. The third and fourth columns show the numbers of fan-in and fan-out cells of each cell, respectively; the number of cells in the other chains is shown on the left side of the slash (/), whereas the number of cells in the defective chain is shown on the right side of the slash (/). As presented in Table 8, the accuracy of stimulus failure prediction is high when a fault occurs in Cell 0. However, the accuracy decreases as a defective cell approaches the scan-out cell. Subsequently, it increases when the fault occurs at the scan-out cell. The correlation between the accuracy and the numbers of fan-in and fan-out cells is insignificant. However, if the number of fan-in cells is 0, the accuracy decreases rapidly, as shown by Cell 11. Therefore, if a cell has no fan-in cells, it must be located at the beginning of the chain. This reordering can increase the accuracy of scan-chain diagnosis.

Next, the correlation between the magnitude of the same fault effect between different faults in the chain, the magnitude of the failure effect of a cell in the chain, and the accuracy are analyzed. If the failure tendency of each cell in the same scan chain is similar, it is difficult to find the defective cell. Suppose there are two scan chains of three cells. The failure effect of the first cell on a chain is the first and second cells of another chain. The failure effect of the second cell on the chain is also the first and second cells of another chain. In this case, the exact defective scan cell cannot be clearly distinguished by any scan-chain diagnosis.

It is also difficult to predict the defective cell if the failure of a cell is not affected anywhere. Suppose the failure effect of the third cell does not affect any cell. The failure on the third cell of the chain does not make any failure on another chain. Therefore, the exact defective scan cell cannot be clearly distinguished by any scan-chain diagnosis.

To measure the magnitude of the same fault effect between different faults in the chain and the magnitude of the failure effect of a cell in the chain, the fan-out similarity is proposed, as well as fan-in similarity, average number of fan-out, and average number of fan-in. Fan-out and fan-in similarity are a measure of how many cells in the same chain share the same cells as the fan-out and fan-in cells of a specified cell. The similarity between Cell A and Cell B (*SIM_AB_*) is defined as follows:

(6) $$SIM_{AB}=\;\frac{N_{AB}}{N_{A}}$$

where *N_A_* is the number of fan-out cells of Cell A and *N_AB_* is the number of the same fan-out cells between Cell A and Cell B. Then, the similarity of a chain is the value that totalizes the similarity of all cells in the chain except the similarity of itself such as *SIM_AA_*. Therefore, the similarity of the chain (*SIM_chain_*) is defined as follows.

(7) $$SIM_{chain}=\;\sum_{i=0}^{n}\sum_{j=0}^{n}SIM_{ij}-\;\sum_{i=0}^{n}SIM_{ii}$$

where *n* is the number of cells in the scan chain. Let us explain with an example. Suppose there are two scan chains of three cells. The fan-out cells of the first cell for the first chain are the first and second cells of another chain; the fan-out cells of the second cell are the second cells of another chain. Finally, the fan-out cell of the third cell is the first cell in another chain. In this case, the fan-out similarity between cells are measured to *SIM_12_* = ½; *SIM_13_* = ½; *SIM*_21_ = 1/1; *SIM*_23_ = 0; *SIM_31_* = 1/1; *SIM_32_* = 0/1. Thus, in this case, the fan-out similarity of the first chain is 3. Likewise, the fan-in similarity is also measured.

The average number of fan-out, the average number of fan-in is a measure of the magnitude of the failure effect of a cell in the chain. In the below example, the total number of fan-out cells of chain 1 is 4, and the number of scan cells is 3. Therefore, the average number of fan-out is 4/3. Likewise, the average number of fan-in is also measured.

Table 9 lists the fan-out and fan-in similarity, the numbers of fan-out and fan-in cells, and the accuracy of scan-chain diagnosis of each chain. The first and second columns show the chain number and accuracy of each scan chain, respectively. The third and fourth columns show the fan-out and fan-in similarity in the corresponding chain, respectively. The fifth and sixth columns show the average numbers of fan-in and fan-out cells in the corresponding chain, respectively.

The scan-chain diagnosis accuracy increased with the average number of fan-in and decreased with the fan-out similarity as shown in Table 9, the average numbers of fan-ins of Chains 8 and 9 in B12 are higher than that of Chain 1, but the fan-out similarities of the two chains are higher than that of Chain 1, therefore, the accuracies of Chains 8 and 9 are less than that of Chain 1. In addition, the lowest scan chain of B14 has the lowest average fan-in cells and the highest fan-out similarity. Therefore, Table 9 shows that the accuracy increases if the effect of the failure spreads more and decreases if the magnitude of the same fault effect between different faults is high.

The scan chains were reordered for increasing the number of fan-in cells in the other chains, to improve the accuracy of scan-chain diagnosis using machine learning. Subsequently, cells that had no fan-in cells were placed at the beginning of the chain. The scan-chain reordering method in these analyses can increase the accuracy of scan-chain diagnoses.

## 5. Conclusions

Machine learning has recently emerged as an important tool for integrated circuit testing; it possesses several features suitable for practical classification forecasting applications. Scan-chain diagnosis is a typical classification problem that can achieve high accuracy for big data in integrated circuit (IC) testing. However, training and testing are difficult to perform due to the extremely long failure log datasets.

Novel fan-in and fan-out filters that highlight failure features and remove unnecessary features in a failure log dataset were proposed herein. These filters can not only reduce the lengths of the input vectors and the size of the models, but also increase the accuracy of scan-chain diagnosis.

The experimental results indicated that the proposed scan-chain diagnosis can achieve a higher accuracy than the previous scan-chain diagnosis with ANN. Specifically, the proposed scan-chain diagnosis achieved an improvement of 3.17% and reduced the construction time by 79.80%, compared with the previous diagnosis. Furthermore, it reduced the diagnosis time by 99.9984%. Therefore, the proposed scan-chain diagnosis is applicable to industrial circuits to achieve the time-to-market goal by reducing the training and diagnosis times.

Henceforth, we will propose an appropriate scan-chain reordering method to obtain a higher accuracy of scan-chain diagnosis. We will also study the multiple scan-chain diagnosis with ML for multiple scan-chain faults in the manufacturing process.

## Figures and Tables

**Table 1:** Fan-in connected cells of example in Figure 6.

Cell	Fan-In Connected Cells
S_1_C_1_	S_2_C_1_, S_2_C_3_, and S_2_C_6_
S_1_C_2_	S_2_C_5_
S_1_C_6_	S_2_C_4_, S2C_5_, and S_2_C_7_
S_3_C_1_	S_2_C_2_ and S_2_C_5_
S_3_C_2_	S_2_C_5_ and S_2_C_6_
S_3_C_6_	S_2_C_7_

**Table 2:** Fan-out connected cells of example in Figure 7.

Cell	Fan-Out Connected Cells
S_2_C_1_	
S_2_C_2_	S_2_C_3_
S_2_C_3_	S_1_C_2_ and S_2_C_3_
S_2_C_4_	S_3_C_5_
S_2_C_5_	S_2_C_3_, S_1_C_4_, S_1_C_7_, and S_3_C_7_
S_2_C_6_	S_3_C_2_, S_3_C_4_, and S_3_C_5_
S_2_C_7_	S_1_C_7_, S_2_C_7_, and S_3_C_7_

**Table 3:** Appropriate solvers for each case.

Case	Solver
L1 penalty	“liblinear” or “saga”
Multinomial Loss	“lbfgs,” “sag,” “saga,” or “newton-cg”
Very Large dataset	“sag” or “saga”

**Table 4:** Characteristics of benchmark circuits.

Circuit	Gate Count	Number of Scan Cells	Number of Scan Chains	Fan-In	Fan-Out
B12	1000	120	10	37/0	65/1
B14	3461	220	10	183/0	220/0
B15	6931	420	10	235/3	326/0
B17	21,191	1320	10	243/2	371/0
B20	7931	430	10	211/0	288/0
B22	12,128	620	10	227/0	285/0
AES	10,656	554	10	35/0	128/0
OR1200	16,637	3420	30	313/0	2905/0

**Table 5:** Accuracy of scan-chain diagnosis for SA0 faults occurring in the first scan chain.

Circuit	Prediction Accuracy (%)		
	[31]	[33]	Proposed Method
B12	83.70%	79.00%	81.60%
B14	24.90%	88.40%	96.24%
B15	93.80%	80.30%	98.62%
B17	61.30%	85.20%	83.93%
B20	95.30%	97.50%	86.49%
B22	75.60%	91.50%	94.01%
AES	-	-	90.06%
OR1200	-	-	82.36%

**Table 6:** Model construction time.

Circuit	Model Construction Time (h)			
	[33]	Filter Gen. Time	Proposed Method	Reduction Ratio
B12	0.10	2.8 × 10^−4^	0.08	20.00%
B14	2.70	9.2 × 10^−2^	0.21	92.22%
B15	4.30	5.5 × 10^−4^	0.76	82.32%
B17	71.60	2.8 × 10^−2^	1.40	98.04%
B20	3.40	9.2 × 10^−2^	0.38	88.82%
B22	25.90	1.5 × 10^−1^	0.68	97.37%
AES	-	1.5 × 10^−2^	0.18	-
OR1200	-	9.5 × 10^−2^	9.05	-

**Table 7:** Diagnosis time.

Circuit	Diagnosis Time (ms)		
	[33]	Proposed Method	Reduction Ratio
B12	48,000	0.89	99.9981%
B14	181,500	2.00	99.9989%
B15	231,000	4.00	99.9983%
B17	3,663,000	48.85	99.9987%
B20	290,250	8.93	99.9969%
B22	1,224,500	7.98	99.9993%
AES	-	3.9	-
OR1200	-	45.77	-

**Table 8:** Accuracy of scan-chain diagnosis for SA0 faults occurring in the first scan-chain cells.

Cell	Cell Accuracy	Fan-Out Cells	Fan-In Cells
Cell 0	86.64%	65/1	7/1
Cell 1	87.62%	66/1	7/2
Cell 2	70.95%	66/1	7/2
Cell 3	64.34%	68/1	7/2
Cell 4	62.13%	66/1	7/2
Cell 5	56.50%	31/6	16/7
Cell 6	49.39%	31/6	16/6
Cell 7	44.24%	31/6	16/6
Cell 8	53.06%	31/6	16/6
Cell 9	49.75%	31/6	16/6
Cell 10	39.59%	31/6	16/6
Cell 11	11.76%	1/1	0/0
Cell 12	56.00%	1/1	0/2

**Table 9:** Accuracy of scan-chain diagnosis for SA0 faults with the fan-out similarity, fan-in similarity, average fan-out cells, and average fan-in cells.

Chain	Scan-Chain Diagnosis Accuracy	Fan-Out Similarity	Fan-In Similarity	Average Fan-Out Cells	Average Fan-In Cells
B12.Chain 0	77.74%	74.27	51.70	13.76923	43.61538
B12.Chain 1	87.12%	36.62	40.00	18.58333	27.25
B12.Chain 2	63.89%	51.77	34.15	14.66667	16.41667
B12.Chain 3	47.85%	97.11	30	9	2
B12.Chain 4	48.61%	97.11	30	9	2
B12.Chain 5	50.88%	98.11	30	8.916667	2
B12.Chain 6	48.23%	97.11	30	9	2
B12.Chain 7	50.51%	81.95	25	10.41667	1.833333
B12.Chain 8	71.97%	62.15	26.11	17.91667	16.08333
B12.Chain 9	80.30%	93.03	31.77	21.33333	9.25
B14.Chain 0	96.24%	582.68	457.12	229.52	20.28
B14.Chain 1	100%	191.59	504.86	71.2	45.76
B14.Chain 2	100%	458.33	546.60	111	39.56
B14.Chain 3	100%	89.29	524.10	51	39.44
B14.Chain 4	100%	40.03	434.03	33.84	63.36
B14.Chain 5	100%	415.03	499.50	54.72	54.72
B14.Chain 6	100%	452.94	486.34	93.36	118.96
B14.Chain 7	100%	404.59	473.91	82.52	128.84
B14.Chain 8	100%	393.20	478.06	69.28	139.08
B14.Chain 9	100%	394.05	446.78	74.12	130.64
